# Efficacy of Clinpro^TM^XT Varnish surrounding brackets on the enamel surface of white spot lesion.

**DOI:** 10.1590/0103-6440202305405

**Published:** 2023-10-27

**Authors:** Ana Zilda Nazar Bergamo, Lucas Portilho Miguel, Renata Zoraida Rizental Delgado, Regina Guenka Palma-Dibb, Juliana Jendiroba Faraoni, Patricia Gatón-Hernández, Raquel Assed Bezerra da Silva, Léa Assed Bezerra da Silva, Paulo Nelson-Filho

**Affiliations:** 1 Department of Pediatric Dentistry, School of Dentistry of Ribeirão Preto, University of São Paulo, SP, Brazil.; 2 Department of Restorative Dentistry, School of Dentistry of Ribeirão Preto, University of São Paulo, SP, Brazil.; 3 School of Dentistry, University of Barcelona, Barcelona, Spain

**Keywords:** White spot, dental enamel, microscopy confocal, fluorides topical, orthodontic brackets

## Abstract

Orthodontics patients usual develop demineralization and present cavity caries lesions after six months. Minimally invasive procedures have been the goal in modern dental practice. The aim of this study was to evaluate the effect of Clinpro^TM^XT Varnish, on the enamel surface roughness and severity of white spot lesions. Twenty premolars were submitted to bond brackets and experimental induction of demineralization and randomly divided into 2 groups: GI - fluoride varnish (Colgate Duraphat®); GII - Ionomeric Sealant (Clinpro^TM^XT Varnish). The treatment was applied around the brackets. The surface roughness of specimens was analyzed, before treatment and 12 weeks after treatment by laser confocal microscopy, and the severity of the white spot lesion was by laser fluorescence device. The data were analyzed by non-parametric Wilcoxon and Mann-Whitney Test, at 5% significance, roughness percentage reduction was performed. The severity of demineralization decreased in both, GI (p = 0.005) and GII (p = 0.019). Enamel superficial roughness levels decreased in GI and GII. As well as the roughness percentage, being more expressive in the Clinpro^TM^XT Varnish group (85,09%). Colgate Duraphat® or Clinpro™ XT Varnish reduced the severity of the demineralization and decreased the superficial roughness on the enamel. The Clinpro™ XT Varnish was superior to superficial roughness on enamel.

## Resumo

Pacientes ortodônticos geralmente desenvolvem desmineralização e apresentam lesões de cárie após seis meses de tratamento. Procedimentos minimamente invasivos têm sido o objetivo na prática odontológica moderna. O objetivo deste estudo foi avaliar o efeito do Clinpro^TM^ XT selante ionomérico, ao redor do bráquete, com relação a rugosidade superficial do esmalte e a severidade da lesão induzida. Vinte pré-molares foram submetidos a colagem de bráquetes e indução experimental de desmineralização e divididos aleatoriamente em 2 grupos: GI - verniz fluoretado (Colgate Duraphat®); GII - Selante Ionomérico (Clinpro^TM^ XT). O tratamento foi aplicado ao redor dos bráquetes. A rugosidade da superfície dos espécimes foi analisada, antes do tratamento e 12 semanas após o tratamento por microscopia confocal a laser e severidade da lesão de mancha branca por dispositivo de fluorescência a laser. Os dados foram analisados pelo teste não paramétrico de Wilcoxon e Mann-Whitney, a 5% de significância. A taxa de redução da lesão foi calculada. A severidade da desmineralização diminuiu tanto no GI (p = 0,005) quanto no GII (p = 0,019). Os níveis de rugosidade superficial do esmalte diminuíram no GI e GII, assim como o percentual de rugosidade, sendo mais expressivo no grupo Clinpro^TM^XT (85,09%). Colgate Duraphat® e Clinpro™ XT reduziram a severidade da desmineralização e diminuíram a rugosidade superficial do esmalte. O selante ionomérico Clinpro™ XT foi superior na redução percentual de rugosidade.

## Introduction

Oral health is the goal in the dental practice. For this, preventive and minimal intervention procedures have been recommended^1^. Despite of this recommendation, noncavitated carious lesions and demineralization have been a growing problem ^2^ ^,^ ^3^ ^,^ ^4^. Patients undergoing orthodontic treatment frequently develop white spot lesions (WSL) at an early stage of the treatment and present cavity caries lesions six months ^5^ after bonding brackets, which has a negative impact on both health and esthetics ^6^.

Several remineralizing treatments have been proposed for the treatment of white spot lesions. Fluoride varnishes, topical fluoride and casein phosphopeptide amorphous calcium phosphate (CPP-ACP) are the most common clinical treatments ^7^ ^,^ ^8^.

During the orthodontic treatment, the ionomeric sealant Clinpro^TM^ XT can be used as a remineralizer like fluoride varnishes. The manufacturer has claimed as an advantage of this material that it remains adhered to the enamel for about 6 months, protecting exposed tooth surfaces from demineralization. It is capable of releasing fluoride, calcium, and phosphate, in addition to being aesthetically imperceptible ^9^ ^,^ ^10^ ^,^ ^11^. The literature describes the effectiveness of Clinpro^TM^ XT Varnish in the prevention of white spot lesions ^11^ ^,^ ^12^. However, that isn’t efficient in restoring of enamel color ^12^ demonstrating a lack of agreement in the literature regarding the effectiveness of this material.

The effectiveness of this material has not been evaluated regarding the reduction of severity and roughness of white spot lesions when applied around orthodontic brackets that should be used during the orthodontic treatment.

Thus, the aim of this *ex vivo* study was to evaluate the effect of a single application of the Clinpro^TM^ XT Varnish - ionomeric sealant on the enamel surface roughness and severity of white spot lesions, induced around orthodontic brackets using fluoride varnish (Colgate Duraphat®) as control. The enamel surface roughness and severity of WSL were determined by confocal microscopy and laser fluorescence respectively.

## Materials and methods

The Research Ethics Committee of Ribeirão Preto School of Dentistry- USP (Process #CAAE 90723118.0.0000.5419), approved the protocol of this research.

The sample size calculation was based on the results of a previous study ^(^ ^13^ resulting in the requirement of 9 specimens per group (Two Tails, effect size 1.37; α prob 0.05; Power 0.95). However, due to the possibility of losses, 10 specimens were used per group.

Twenty recently human extracted teeth (premolar), lesion-free, obtained from the Human Teeth Biobank were selected. Visual examination using a magnifying stereoscope with a 10x magnification (Carl Zeiss, Jena, Germany) and clinical examination with an explorer probe were done. Teeth with cracks, fractures, caries lesions, or structural anomalies were excluded from the sample. The teeth were then randomly assigned into two groups, according to the different treatments:

- Group I: Topical single application of Colgate Duraphat^®^ fluoride varnish (Professional^®^; Colgate-Palmolive Indústria e Comércio Ltda., São Bernardo do Campo, SP, Brazil) on the enamel around orthodontic brackets, with and without induction of white spot lesion. - Group II: Single application of Ionomeric Sealant Clinpro™ XT Varnish (3M do Brasil Ltda., Sumaré, SP, Brazil) on the enamel around the orthodontic brackets, with and without induction of white spot lesion.

Metal brackets (Slim Max, Morelli Ortodontia, Sorocaba, SP, Brazil) for incisors were positioned in the center of the buccal surface of all specimens and bonded with light-activated orthodontic adhesive (Transbond Adhesive XT; 3M Unitek, Monrovia, CA, USA), according to the manufacturer’s instructions. The bonding was done in two steps: first, a layer of etch-and-rinse adhesive was applied, dried with air for 5 seconds, and photoactivated for 20 seconds. The brackets were positioned, seated firmly on the tooth surface, and resin excess was removed. The adhesive was cured by positioning the angle light guide mesial and distal to each bracket for 20 seconds on each face, totalizing 40s for each bracket.

The radicular portion was removed with a diamond disc mounted on a cutting machine (Minitom - Struers A / S, Copenhagen, Denmark), 2mm below the enamel-cement junction.

The vestibular surface was divided into two areas. One area of 20mm^2^ was covered with adhesive tape (Silver taper red Aldebras, São Paulo Brazil). Then, the teeth were dipped in pink wax no 7 (Wilson-polidental LTDA / Cotia São Paulo, Brazil) to cover the remaining parts and, in sequence, the adhesive tape was removed with the aid of a scalpel blade. Then, the specimens were stored in a demineralizing solution of Calcium Nitrate, containing 1.28mM of Calcium, 0.74mM of Phosphate, and 0.03µg Fluorine / mL, for 43 hours at 37ºC (pH 4.5) ^(^ ^13^. After the remotion of adhesive tape, each specimen presented the following areas: surface A (SA): sound enamel, and surface B (SB): enamel with demineralization.

**Figure 1:**
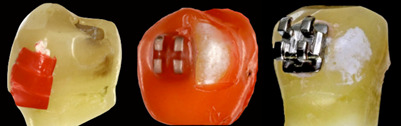
Induction of White spot lesion.

The teeth were fixed in PVC tubes (2cm high x 2.5 cm in diameter) filled with chemically activated acrylic resin (JET - Classic, Campo Limpo Paulista, Brazil), keeping the surface enamel labial facing upwards, for analysis in confocal microscopy and laser fluorescence. Throughout the experimental period, the specimens were stored in artificial saliva (KH_2_PO4, K_2_HPO4, KCl, NaCl, MgCl_2_.6H_2_O, CaCl2._2_H_2_O, NaF, sorbitol, nipagin, nipasol, carboxymethylcellulose (CMC), water - pH 7.0) ^17^, which was changed weekly and kept at 37°C. (Figure 1)

After the baseline (T0) readings (Laser Fluorescence and Laser confocal microscopy) both the fluoride varnish and the ionomeric sealant were applied with a microbrush around the brackets, following the manufacturers' instructions.

The specimens were reevaluated 12 weeks (T1) after the implementation of the treatments. According to the manufacturer's instructions of Clinpro^TM^XT Varnish, after 12 weeks, the material should be detached, and the area submitted for prophylaxis.

All specimens after 12 weeks have been submitted to prophylaxis with a rubber cup and pumice power.

Before (T0) and after (T1) treatment, the sound enamel (SE) and the WSL vestibular surface were submitted to fluorescence measurements by the device DIAGNOdent model 2095 (KaVo Dental, Biberach/Riß, Germany), using tip B. The calibration of DIAGNOdent has been done as recommended by the manufacturer, against the ceramic standard. The highest reading was recorded on both analyzed sites (sound enamel and enamel with white spot lesions). A single calibrated operator measured the sites using the laser fluorescence device. After drying the sample with air for 2 seconds, the fluorescence of a sound smooth surface of the teeth was assessed. The moment and the peak were recorded, and the average value was used.

Enamel roughness was measured on the vestibular enamel surface using a laser confocal microscope (Lext OLS4000^®^; Olympus, Tokyo, Japan). The specimens were positioned parallel to the table of the microscope, using specific software (OLS40000^®^, Olympus, Japan), with images of the 2 areas (surfaces A and B) in the upper on the right and left side and the bottom of each specimen, with 10x magnification. The initial surface roughness (T0) before treatment and 12 weeks (T1) after the treatment were measured in µm (micrometers), covering an area of approximately 0.5mm^2^ on the surfaces A and B. A single calibrated operator measured all the sites.

Since different rates of demineralization were obtained, and each specimen may differ in roughness at baseline, it has been used for comparison of the reduction tax by this index:

$$Reduction\;roughness\;\left(\%\right)=(\;{meanR}_{b}\;-\;{meanR}_{a}\;x100)/{meanR}_{b}\;$$

Rb roughness before treatment. Ra roughness after treatment.

### Statistical analysis

The scores obtained with the laser fluorescence device were evaluated by the Wilcoxon test for intragroup analysis. In the same way the laser confocal microscopy. Intergroup analysis was performed by the Mann-Whitney Test. The significance level adopted for all analyses was 5%.

## Results

To ensure that the demineralizing process was effective, the severity of the lesion was measured by comparison of the sound enamel with the white spot area in each group, before and after treatment. The T0 values in enamel with demineralization were higher than sound enamel (pGI=0.008; pGII=0.031), which confirmed the effectiveness of the demineralization process. However, no significant statistical difference (T1p>0,05) was observed between SE and WS at T1. Table 1 presents the median, first, and third quartile values.

**Figure 2:**
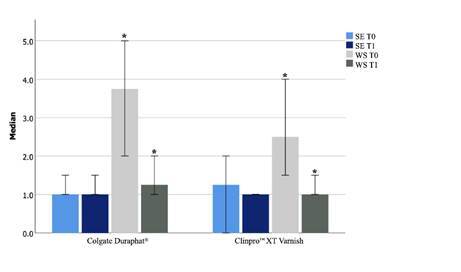
Laser fluorescence evaluation - White spot lesion severity before and 12 weeks after a single application of Colgate Duraphat^®^ and Clinpro™ XT Varnish. GI- Colgate Duraphat^®^; GII- Clinpro™ XT Varnish; T0- before treatment; T1- 12 weeks after treatment; SE- sound enamel; WS- white spot; * statistical significance; pGI=.005; pGII=.019.

The intragroup analysis showed the treatment’s effectiveness, with the decrease in the severity of white spots in both the Colgate Duraphat^®^ and the Clinpro^™^ XT Varnish demonstrated in Figure 2.

The intergroup analysis demonstrated a similarity between both treatment responses, no difference was found between the treatments (p>0.05).

**Table 1:** Fluorescence laser - DIAGNOdent, descriptive analysis

Group	T0-SE _^Median (Q1-Q3)^_	T1-SE _^Median (Q1-Q3)^_	T0-WS _^Median (Q1-Q3)^_	T1- WS _^Median (Q1-Q3)^_
GI	1.00(0.0-1.5)	1.00(1.0-1.05)	3.75 (2.38-5.0)	1.25 (1.00-1.63)
GII	1.25(0.0-2.0)	1.00(1.0-1.0)	2.50 (1.85-1.50)	1.00 (1.00-1.50)

GI- Colgate Duraphat^®^; GII- Clinpro™ XT Varnish; T0 - Before treatment; T1 -12 weeks after treatment; SE sound enamel; WS white spot; Q1- first quartile; Q3- third quartile.

**Figure 3:**
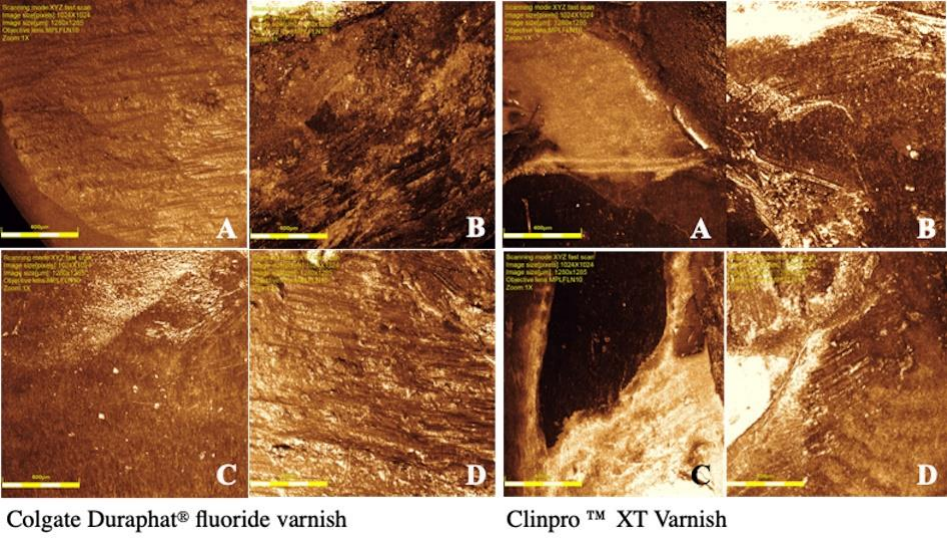
Confocal laser microscopy representative images of superficial roughness obtained on sound enamel and white spot lesion before and 12 weeks after a single application of Colgate Duraphat^®^ and Clinpro™ XT Varnish. A-sound enamel before treatment; B-sound enamel after treatment; C-white spot lesion before treatment; D-white spot lesion after treatment.

The roughness was evaluated, also, at T0 and T1, table 2 contained a descriptive analysis of the data. The values were higher in the white spot area than in sound enamel, for both groups Colgate Duraphat^®^ (p=0.009) and Clinpro™ XT Varnish (p=0.036). Figure 3 shows representative images of superficial roughness confocal microscopy for both groups.

**Table 2:** Confocal Microscopy - descriptive analysis

Group	T0-SE Median (Q1-Q3)	T1-SE Median (Q1-Q3)	T0-WS Median (Q1-Q3)	T1- WS Median (Q1-Q3)
GI	1.65 (1.34-1.84)	1.90 (1.80-2.00)	2.63(1.76-4.94)	1.58 (1.29-2.42)
GII	1.47 (1.23-1.65)	2.17(1.85-2.57)	1.86 (141-2.54)	1.62 (1.25-2.69)

GI- Colgate Duraphat^®^; GII- Clinpro™ XT Varnish; T0 - Before treatment; T1 -12 weeks after treatment; SE sound enamel; WS white spot; Q1- first quartile; Q3- third quartile.

There was a decrease in the enamel superficial roughness in the Colgate Duraphat® and in the Clinpro™ XT Varnish group on demineralized areas; nevertheless, no statically significance was observed (p>0,05). No difference was observed when comparing the two treatments (intergroup p>0.05)

However, it was observed a roughness percentage reduction on enamel with demineralization more expressive in the Clinpro™ XT Varnish group (85,09%), in comparison to the Colgate Duraphat® group (46.97%).

## Discussion

Several studies have shown that fluoride varnish decreases enamel solubility and remains effective for long periods ^14^ ^,^ ^15^. Alternatively, Clinpro^TM^ XT Vanish ionomeric sealant is an available material in the specialized market that releases fluoride, calcium, and phosphorus controlled ^7^, at this moment there are few studies in orthodontic area ^8^ ^,^ ^9^ ^,^ ^11^. For this reason, the present study aimed to evaluate the effectiveness of Clinpro^TM^ XT Vanish which could potentially be used during orthodontic treatment to prevent the development of white spot lesions in orthodontic practice, using an ex vivo laboratory method, that is a reliable model to assess potential remineralizing agents.

The crystalline structure of hydroxy apatite facilitates cationic and anionic substitutions, being referred to as capable of incorporating Mg^2+^, CO3^2−^, Fe^2+^, and F^-^, among others. The focal point of these ionic exchanges is based on the epitaxial deposition of calcium and phosphate ions on existing apatite crystallites. Many materials, including fluoride varnishes such as Duraphat, glass ionomer-based materials, Regenerate, infiltrating resins and MI paste promote enamel remineralization^16^ ^,^ ^17^ ^,^ ^18^ ^,^ ^19^ ^,^ ^20^. These materials release calcium and fluoride ions that promote the obliteration of pores^16^ and regularization of the enamel surface. On the other hand, orthodontic treatment demands a long time of intervention. Then, a material with one application, such as the Clinpro^TM^XT Varnish, and effective for a long time would be ideal in white spot lesion prevention.

Few studies published in the specific literature compared Duraphat varnish and Clinpro^TM^XT Varnish in the treatment of white spots. Escobar-García ^20^ demonstrated that Duraphat^®^ varnish and Clinpro^TM^ White Varnish are effective in the remineralization of white spot lesions, but Duraphat^®^ varnish was less biocompatible. Lena Sezici ^(^ ^19^ observed that Clinpro^TM^ Varnish was more effective than Duraphat^®^ varnish, diminishing the fluorescence loss. Shah ^21^ related that one single application of Clinpro^TM^Vanish ionomeric sealant could prevent enamel demineralization for a longer period (4 months) when compared with Duraphat^®^ varnish, whose effectiveness will last for 45 days. However, the Clinpro^TM^XT Vanish ionomeric sealant advantage over fluoride vanish is it being aesthetically imperceptible and fluoride release for six months, protecting teeth surfaces from demineralization ^(^ ^15^.

In the present study, was observed a reduction in the lesion severity by laser fluorescence analysis in demineralizations, after 12 weeks, in both groups of treatment. Thus, our results confirm the effectiveness of Duraphat^®^ varnish and Clinpro^TM^ XT Varnish ionomeric sealant in remineralization 12 weeks after its application.

Laser fluorescence device has been shown to be an effective and non-destructive diagnostic method for the longitudinal evaluation of superficial caries ^8^ ^,^ ^22^. The results of this research demonstrated a reduction in the severity of WSL, also corroborated by the reduction in percentage surface roughness using confocal microscopy.

 Regarding surface roughness, it was employed confocal laser microscopy is a tool for obtaining high-resolution images, 3D reconstructions, and optical sections using 3D samples ^23^ ^,^ ^24^. Our results showed a decrease in the values of the surface roughness in the white spot area in both groups. However, the roughness percentage reduction was more expressive on in the Clinpro^TM^ XT Vanish ionomeric sealant group.

Finally, we can highlight the positive results of Clinpro^TM^ XT Vanish ionomeric sealant in the reduction of the severity of the demineralization verified by the analysis of the fluorescence diagnosis and an expressive tax of roughness reduction. It has a more intense adhesion when compared to the varnish and will remain active for six months. Thus, the results of this study suggest that ionomeric sealant proved to be a good material to be used in the remission of demineralization in patients undergoing orthodontic treatment.

### Limitations

Clinical studies are necessary to compare the effectiveness of those materials in clinical conditions, such as daily brushing and food intake, to analyze effectiveness through abrasion processes, providing support for the results obtained in the present study ex vivo.

Considering the limitations of ex vivo studies and the results obtained, it was concluded that one single application of Colgate Duraphat® and Clinpro™ XT Varnish reduced the severity of the demineralization and decreased the superficial roughness on enamel with demineralization. The Clinpro™ XT Varnish was superior in roughness percentage reduction. This material presents an interesting potential for clinical use.
